# Bayes optimal template matching for spike sorting – combining fisher discriminant analysis with optimal filtering

**DOI:** 10.1007/s10827-015-0547-7

**Published:** 2015-02-05

**Authors:** Felix Franke, Rodrigo Quian Quiroga, Andreas Hierlemann, Klaus Obermayer

**Affiliations:** 1 ETH Zürich, Department of Biosystems Science and Engineering, Basel, 4058 Switzerland; 2 School for Electrical Engineering and Computer Science, Technische Universität Berlin, Berlin, Germany; 3 Centre for Systems Neuroscience, University of Leicester, Leicester, UK

**Keywords:** Spike sorting, Extracellular recording, Signal processing, Overlap, Linear filtering, Linear discriminant analysis, Matched filtering

## Abstract

Spike sorting, *i.e.*, the separation of the firing activity of different neurons from extracellular measurements, is a crucial but often error-prone step in the analysis of neuronal responses. Usually, three different problems have to be solved: the detection of spikes in the extracellular recordings, the estimation of the number of neurons and their prototypical (template) spike waveforms, and the assignment of individual spikes to those putative neurons. If the template spike waveforms are known, template matching can be used to solve the detection and classification problem. Here, we show that for the colored Gaussian noise case the optimal template matching is given by a form of linear filtering, which can be derived *via* linear discriminant analysis. This provides a Bayesian interpretation for the well-known matched filter output. Moreover, with this approach it is possible to compute a spike detection threshold analytically. The method can be implemented by a linear filter bank derived from the templates, and can be used for online spike sorting of multielectrode recordings. It may also be applicable to detection and classification problems of transient signals in general. Its application significantly decreases the error rate on two publicly available spike-sorting benchmark data sets in comparison to state-of-the-art template matching procedures. Finally, we explore the possibility to resolve overlapping spikes using the template matching outputs and show that they can be resolved with high accuracy.

## Introduction

The detection and classification of voltage deflections in extracellular recordings caused by action potentials of neurons - called spikes - is known as spike sorting. It is necessary if single neuronal activities must be resolved from multi-neuronal firing activity. The assignment of spikes to neurons is only possible because the spike shapes of neurons differ due to their morphology, their spatial position with respect to the recording electrode(s), the intrinsic membrane properties of the neuron and the surrounding medium (Camuñas-Mesa and Quiroga 2013; Gold et al. 2006). Furthermore, at least to a first approximation, spikes from the same neuron have similar waveforms. It is, therefore, possible to group extracellular potentials based on their waveform assuming that spikes within one group were actually emitted by the same neuron.

In principle, extracellular recordings can be seen as having two different linearly added components, background activity (noise and small action potentials from far away neurons) and spikes of close-by cells (Buzsáki 2004; Camuñas-Mesa and Quiroga 2013). Therefore, for any given piece of data, spike sorting typically solves three problems (Einevoll et al. 2012; Lewicki 1998): First, spikes are detected in the noisy recording. Second, spikes are extracted from the data, aligned and their dimensionality is reduced using feature extraction. Third, spikes, which are now represented by a small number of features, are grouped into clusters of similar spike shapes that putatively originate from the same neuron. However, there is another option: Spikes can be assigned to neurons by using knowledge from a preceeding clustering step, which we refer to as spike classification. At first the classification problem seems to be redundant because spikes are assigned to putative neurons already during the clustering step. But there are several reasons why it is important to treat the classification problem separately from the clustering step. Among these, classification is usually much faster than clustering, an important advantage for online applications, where it might be desirable to use a fast classifier that was derived from an initial offline clustering for real-time spike sorting. Additionally, many clustering procedures scale poorly with the number of spikes and their application becomes infeasible for very long recordings. Then, only a subset of spikes can be clustered and the rest simply classified.

One way to build such a classifier is to calculate the average of all elements for each cluster. This cluster center is called the template. Each unclassified spike is then compared to each template and is subsequently assigned to the template that was most similar to it according to some appropriate similarity measure. This procedure is often referred to as template matching.

**Fig. 1:**
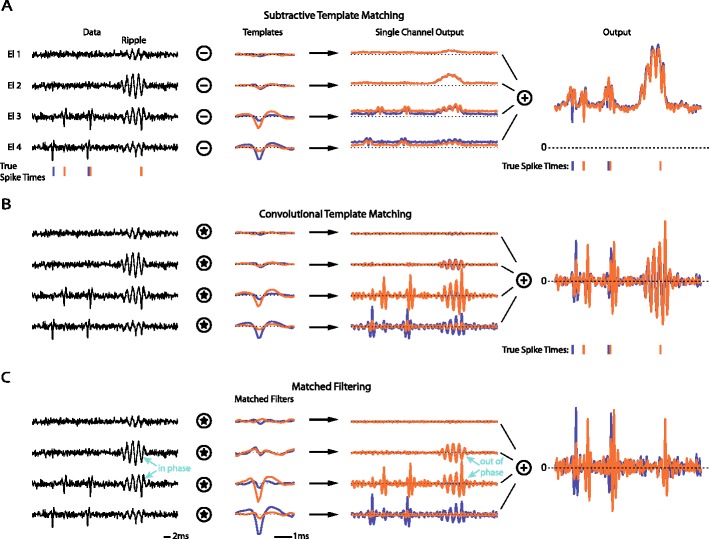
Illustration of different template matching techniques for a toy example (artificial data; y-axis arbitrary units). A short piece of a 4 electrode recording was simulated by copying two templates and a simulated ripple into Gaussian white noise. The first column of panels shows the data, the second column the templates (and derived filters), the third column the respective single electrode template matching outputs, and the last column the final multichannel template matching output. For all three methods spikes would have to be detected in the template matching outputs by thresholding (in **a** with a threshold on minima, for **b** and **c** on maxima of the output). The second to last column shows the contribution of individual electrodes to the final template matching output. **a** Euclidean distance of the data to each template. Known templates are subtracted at each possible position from the data (for each electrode individually) and the norm of the residual (over all electrodes) is computed. If this residual is close to zero, the template is assumed to be present in the data at the respective temporal position. **b** Convolution of the data with the multi-electrode templates. For each multi-electrode template the data from every electrode is convolved with the respective single-electrode template individually and the results are summed. **c** Convolution of the data with matched filters. Matched filters are computed from the template and the noise covariance matrix (in this example including the statistics of the “ripple”)

Here, we focus exclusively on the detection and the classification problem: If the number of neurons and their templates are known, *e.g.*, as the result of an offline spike sorting procedure, what is the best way to perform template matching? Different approaches to solve this problem were proposed (see *e.g.,* (Abeles and Goldstein 1977; Friedman 1968; Gerstein and Clark 1964; Keehn 1966; Salganicoff et al. 1988) but also recent approaches (Vargas-Irwin and Donoghue 2007; Zhang et al. 2004)) including filter based methods (Roberts and Hartline 1975) (see Fig. 1 for an illustration). Although template matching is of great importance for the analysis of extracellular recordings, it was not thoroughly investigated yet what the best strategy is. Even current commercial products rely on very simple strategies like Euclidean distance (Cambridge Electronic Design Limited 2012; Plexon Inc 2009) with manual threshold selection.

**Fig. 2:**
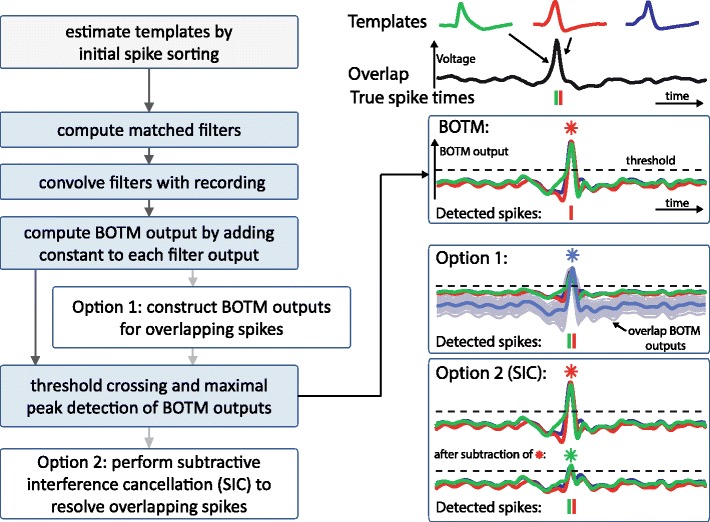
Flowchart of the BOTM method (*left*) and illustration of the most important steps (*right*) on an example overlapping spike from Benchmark 1 (Q) (*top*, *right*). BOTM outputs are computed for each template (box labeled “BOTM”) and thresholded to detect and classify spikes. To resolve overlapping spikes two alternative approaches are possible. Box labeled “Option 1”: from the individual template BOTM outputs more BOTM outputs are constructed that reflect the presence of overlapping spikes (light grey traces on the right, here shown only for overlaps of maximal 2 spikes and 3 samples temporal difference); the one with the highest peak is marked in dark grey). Box labeled “Option 2 (SIC)”: Once a spike is detected by threshold crossing on the BOTM outputs, it is subtracted from each BOTM output and the threshold is reapplied iteratively

**Fig. 3:**
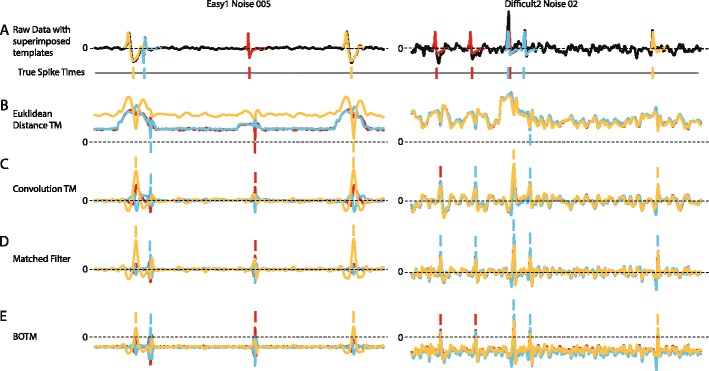
Example of different template matching outputs on a public benchmark data set with a single electrode simulation. **a** Raw simulated data of two different data files from the benchmark. The true spike times (shown as ticks below the data) are known since the data was simulated. At the position of the true spike times the respective templates are superimposed over the data. **b** Output of Euclidean distance template matching. Colored traces correspond to the template matching output of the respective template in **a**. Even on the high signal-to-noise-ratio data file (*left*) not all spikes can be reliably detected by thresholding the output: the first spike of the orange neuron is obscured by the partial overlap. In the low SNR case (*right*) the output cannot be thresholded in a useful way to detect spikes. **c** Convolution-based template matching gives in both cases clear peaks at the positions of the spikes. But not always is the template matching output with the highest peak also associated with the correct template. **d** The matched filter is in this case very close to the convolution-based template matching. But for the difficult data set (*right*) all spikes would be classified as the blue neuron by a simple threshold. **e** Thresholding the BOTM output recovers spike times and their correct identities in both examples (with the exception of the overlapping spike in the difficult data set). This overlap would have to be resolved by a post-processing step. Since the color of the template matching outputs with the highest peak is not always clearly visible, a line with the same color is placed at the respective peaks (maxima in **c**-**e**, minima in **b**) and can be compared to the true spike identities in **a**

**Fig. 4:**
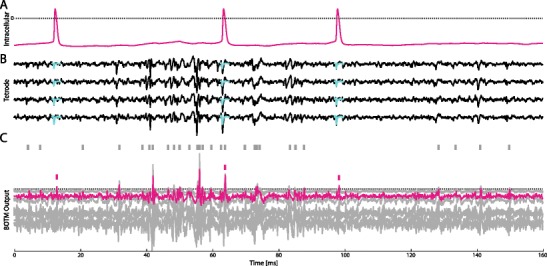
Illustration of the BOTM responses on a 160 ms piece of benchmark 2 (H). **a** Intracellular recording of a single neuron. **b** Extracellular recording using a tetrode located nearby the neuron recorded in **a**. At the time points at which a spike was detected in the intracellular recording the spike-triggered average (template) of the target neuron superimposes the recordings (*cyan traces*) for each electrode. **c** BOTM output and sorted spikes. In this recording, 10 putative neurons were found by the initial spike sorting, one of which was assigned to the target neuron (*magenta*). Each putative neuron has an associated BOTM output. For sake of clarity, neurons for which no ground truth was available are depicted in grey. Small ticks over the traces show the sorting output of the BOTM + SIC procedure. The three spikes of the target neuron were correctly detected and classified. The spike in the middle was classified as part of an overlapping spike. The dotted line represents the detection threshold

In the toy example in Fig. 1 three template matching procedures are illustrated and it can be seen that they respond differently to noise, artifacts (in this case a narrow band oscillation or “ripple”) and overlapping spikes. Convolution (Fig. 1b) has the advantage that noise on electrodes which are too far away to measure the activity of a neuron, and, thus, do not carry information about the presence of the neuron’s template, are suppressed (topmost electrode). Additionally, overlapping spikes produce detectable peaks in the output. However, an artifact or “ripple” may also lead to strong responses. If the noise and ripple characteristics are known, matched filters can be used instead, which have the potential to suppress unwanted signal components. The implementation in this case can be done by using the ripple on the seemingly useless electrode 2 - on which the templates have nearly no energy - to cancel the ripple on electrodes 3 and 4 (marked with “in phase” and “out of phase” respectively). All three methods have the problem that setting the detection threshold is not straight-forward.

Here, we show that template matching can be seen as a filtering operation and propose a finite impulse response (FIR) filter based online template matching procedure that is - under the additional assumption of Gaussian noise - optimal in a Bayesian sense. We derive its optimality (to discriminate spikes from different neurons and to detect the spikes in noisy recordings) from Fisher’s linear discriminant analysis (LDA) and show that this way the outputs of matched filters can be interpreted in a Bayesian sense. The proposed Bayes optimal template matching (BOTM) computes the linear discriminant functions by a convolution of FIR filters with extracellular data and solves both the detection and the classification problem. In contrast to the other methods, BOTM provides an optimal detection threshold analytically and does not require manual intervention. Using BOTM to simultaneously detect and classify spikes removes the need for spike alignment since the peak of the detector output is a robust estimate of the true position of the spike.

We evaluate our method on two previously published benchmark data sets and show that we can significantly improve template matching performance in both cases. Additionally, using BOTM as a post-processing step after clustering can reduce the number of errors which occurred during the initial spike sorting step.

This framework is then extended to overlapping spikes. Detecting and resolving overlapping spikes is an important problem for spike sorting: waveforms of near coincident action potentials will interfere with each other, severely altering the individual waveforms and effecting their detection and classification. Approaches to solve this problem were suggested (Atiya 1992; Ekanadham et al. 2014; Franke et al. 2010; Lewicki 1994; Marre et al. 2012; Pillow et al. 2013; Prentice et al. 2011; Segev et al. 2004; Vargas-Irwin and Donoghue 2007; Wang et al. 2006; Zhang et al. 2004) but are in general computationally expensive. Here, we show that resolving overlapping spikes in the linear discriminant function space could provide a computationally efficient alternative.

Due to its computational simplicity BOTM is especially useful for online and real-time implementation in the context of closed-loop experiments after the initial templates have been found (Einevoll et al. 2012; Franke 2011; Franke et al. 2010; Obeid et al. 2004; Rutishauser et al. 2006).

## Method

### Definitions

Spike sorting relies on the assumption that the action potentials of a single neuron lead to extracellular spikes with similar waveforms (Lewicki 1998). This is generally not true, since spikes of the very same neuron are known to vary, *e.g.*, dependent on the recent firing history of the neuron (Fee et al. 1996) and slowly over time (Franke et al. 2010). Here we will assume non-varying waveforms but, in principle, the filters could be adapted over time. We define the template of neuron *i* as ***ξ***_*i*_ = [*ξ*_*i*,1_^*T*^ , *ξ*_*i*,2_^*T*^, …, *ξ*_*i*,*N*_^*T*^]^*T*^ where *ξ*_*i*,*c*_ is the waveform for neuron *i* on electrode *c* and (·)^*T*^ is the vector transpose. The single electrode waveforms are *L* samples long. Thus ***ξ***_*i*_ is a column vector with *LN* dimensions, where *N* is the number of recording electrodes, *i.e.*, the single electrode waveforms are concatenated. We use an analogous definition for any piece of multi-electrode data starting at time *t*: *X*(*t*) = [***x***(*t*)_1_^*T*^, ***x***(*t*)_2_^*T*^, …, ***x***(*t*)_*N*_^*T*^]^*T*^, where ***x***(*t*)_*k*_ = [*x*(*t*)_*k*_, *x*(*t* + 1)_*k*_, …, *x*(*t* + *L* − 1)_*k*_]^*T*^ represents *L* samples of recorded data on electrode *k* starting at time *t*. The output of a multielectrode FIR filter ***f*** = [*f*_1_^*T*^, *f*_2_^*T*^, …, *f*_*N*_^*T*^]^*T*^ applied to the recordings (which is usually denoted as $y={\displaystyle \sum_k^N}\left({x}_k\star {f}_k\right)$ where ⋆ is the cross-correlation between data of electrode *k* and the filter for electrode *k*) can now be expressed in terms of a vector multiplication: *y*(*t*) = *X*(*t*)^*T*^ ***f***. Note that ***ξ***_*i*_, ***f*** and *X*(*t*) are all column vectors in the same *LN* dimensional vector space.

The noise covariance matrix (Pouzat et al. 2002) is given by: ***C*** = *E*[*η*(*t*)*η*(*t*)^*T*^] with *η*(*t*) being a piece of data where no spikes were detected. It characterizes noise from various sources, including small amplitude spikes from far away neurons. Thus ***C*** is of dimensions *LN* × *LN* and captures the complete spatio-temporal covariance (between electrodes and over time) that is induced by both, noise and undetected small amplitude spikes (Pouzat et al. 2002). Since we assume that the data was high pass filtered *E*[*η*(*t*)] = 0 holds.

### Classical template matching

Two different similarity measures between a piece of data and a template are commonly employed for template matching: Euclidean distance template matching (*e.g.*, (Cambridge Electronic Design Limited 2012; Plexon Inc 2009; Sato et al. 2007)) and convolution or cross-correlation template matching (Friedman 1968; Kim and McNames 2007). Note that convolution and cross-correlation are identical, apart from the time reversal of the filter by the convolution. Although we use the cross-correlation throughout this work, we still refer to template matching based on the cross-correlation as convolutive template matching. The Euclidean distance at time *t* between data *X*(*t*) and template *ξ*_*i*_ is defined as

$$ {D}_i^{Euc}(t)={\left|\left|X(t)-{\xi}_i\right|\right|}^2={\left(X(t)-{\xi}_i\right)}^T\left(X(t)-{\xi}_i\right) = X{(t)}^TX(t)-2X{(t)}^T{\xi}_i+{\xi_i}^T{\xi}_i $$

and the cross-correlation as

$$ {D}_i^{XC}(t)=X{(t)}^T{\xi}_i. $$

### Template matching using the noise covariance matrix

If the noise covariance matrix is known, the recorded data can be prewhitened (Pouzat et al. 2002; Rutishauser et al. 2006) before matching the templates. Prewhitening is a linear operation that transforms the cluster of “noise” waveforms in a way that it will be roughly spherical (standard normal distributed) after prewhitening. With respect to template matching, prewhitening is equivalent to using the squared Mahalanobis distance instead of Euclidean distance and the matched filtering procedure instead of the convolution with the template:

$$ {D}_i^{Maha}(t)={\left(X(t)-{\xi}_i\right)}^T{\boldsymbol{C}}^{-1}\left(X(t)-{\xi}_i\right) = X{(t)}^T{\boldsymbol{C}}^{-1}X(t)-2X{(t)}^T{\boldsymbol{C}}^{-1}{\xi}_i+{\xi_i}^T{\boldsymbol{C}}^{-1}{\xi}_i $$

$$ {D}_i^{Match}(t)=X{(t)}^T{\boldsymbol{C}}^{-1}{\xi}_i=X{(t)}^T{\boldsymbol{f}}_i $$

where ***f***_*i*_ = ***C*** ^− 1^ *ξ*_*i*_ is the matched filter (see the appendix for a more detailed explanation) for template *ξ*_*i*_. Figure 1 illustrates different template matching techniques applied to a toy example. We will refer to *D*_*i*_^*Euc*^ or *D*_*i*_^*Maha*^ as subtractive template matching since templates are subtracted from the data and the energy of the residual is used for spike detection and classification. Accordingly, *D*_*i*_^*XC*^ and *D*_*i*_^*Match*^ are referred to as convolutive template matching.

### Bayes optimal template matching (BOTM)

**Fig. 5:**
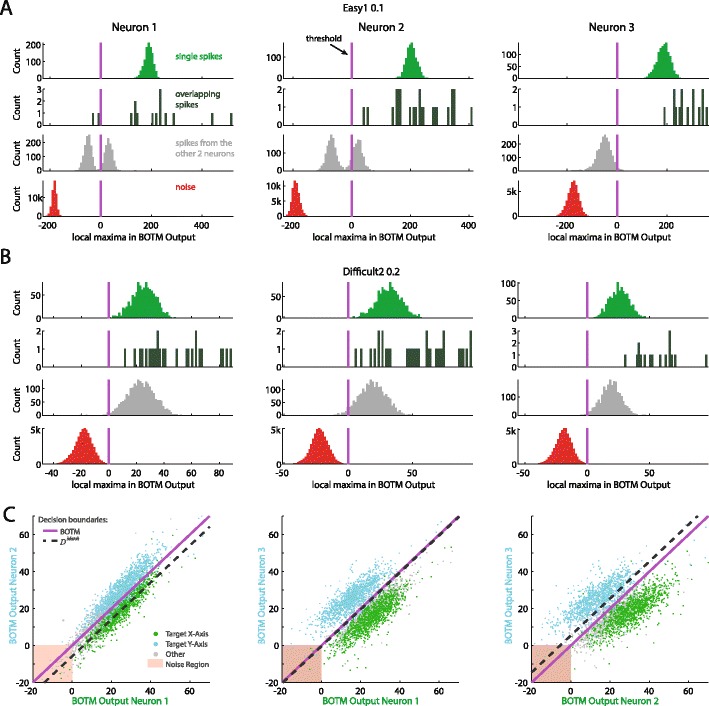
Distributions of local maxima (in a window of 15 samples) in BOTM filter outputs for single spikes, overlaps (within 15 samples), and noise for the three neurons in benchmark 1 (Q) Easy1 N.10 (**a**) and Difficult 2 N.20 (**b**–**c**). The optimal threshold *thr* for p(n) = 0.99 is shown as a vertical line (magenta). For each neuron, spikes from other neurons (second row from bottom, grey) can cause filter responses to cross the threshold. Thus, spike classification can only be done reliably by combining the information from all three BOTM outputs. **a**-**b** The majority of overlapping spikes (second row from top, dark green) causes the BOTM outputs of all participating neurons to cross threshold. Please note that with *thr* = ln(*p*(*n*)), for this example, the exact choice of p(n) does not strongly influence sensitivity: for a wide range of choices for p(n) the threshold will be close to zero which will separate the noise from the spike distribution. **a** For all three neurons the response to noise (bottom, red) is well separated from the response to the target spikes (top, green) by a large margin. Spike detection and classification of single spikes can be done without error (except for overlapping spikes) since the green, grey and red distributions do not overlap. **b** In the low SNR case the green and red distribution are close to each other and start overlapping. Note that the overlap between the “single spike” distribution and the “other spikes” distribution does not directly imply any classification errors, since the classification depends on the maximal response over all three neurons. **c** Projection of the spikes from **b** in the response space spanned by two of the three BOTM outputs (all three combinations are shown). In this space, the discrimination boundary given by a max-operation on the BOTM outputs is the identity line (magenta). Spikes are colored according to their ground truth identity: spikes that should elicit the strongest response for the BOTM output on the x-axis are shown in green; spikes that should elicit the strongest response for the BOTM output shown on the y-axis, and, therefore, should lie above the identity line, are shown in cyan. Spikes of the neuron for which the BOTM output is not shown are grey. If all BOTM outputs are below the threshold (0) they are not detected (‘noise region’). For comparison, the respective decision boundary of *D*_*i*_^*Match*^ is shown as a dashed line

Template matching has to solve two tasks: the detection of a known signal in a noisy recording and the discrimination of spikes originating from different neurons. In the Gaussian noise case, these two problems have known solutions (see appendix): the optimal linear detector (*i.e.*, the linear filter that maximizes the signal-to-noise ratio after filtering) is given by matched filtering (Van Trees 2002), while the optimal discrimination between several clusters that share the same covariance matrix is given by linear discriminant analysis (LDA) (Fisher 1936). These two solutions are interrelated, and, by using the probabilistic framework of LDA, it can be shown that a template matching exists that is optimal with respect to both tasks, *i.e.*, it maximizes the signal-to-noise ratio for each neuron while also maximizing their discriminability. This Bayes optimal template matcher (BOTM) is given by:

$$ {D}_i^{BOTM}(t)=X{(t)}^T{\boldsymbol{C}}^{-1}{\xi}_i-\frac{1}{2}{\xi_i}^T{\boldsymbol{C}}^{-1}{\xi}_i+ ln\left(p(i)\right)=X{(t)}^T{\boldsymbol{f}}_i-\frac{1}{2}{\xi_i}^T{\boldsymbol{f}}_i+ ln\left(p(i)\right) $$

where *p*(*i*) is the prior probability to observe a spike of neuron *i*. For a derivation see eq. (7) in the appendix. In this work we set the prior probabilities equal to a constant (see section”Evaluation metrics“and Fig. 5), although, in principle, the estimated firing rates of the individual neurons could be used. The detection threshold for a spike in the BOTM filter outputs *D*_*i*_^*BOTM*^(*t*) (see Fig. 5) is then given by (see eq. (9) in the appendix)

$$ thr= \ln \left(1-{\displaystyle \sum_i}p(i)\right) $$

which is usually close to 0 for realistic spike priors (see Fig. 3 bottom row).

BOTM computes matched filter outputs for each template (see Fig. 4 for an example with 10 templates). For each template a constant, which depends on the energy (*ξ*_*i*_^*T*^ ***C*** ^− 1^ *ξ*_*i*_) of the respective template and on the probability *p*(*i*) that the template occurs in the data, is added to the filter outputs to compute the final discriminant functions. Spikes are detected and classified by thresholding those discriminant functions (the BOTM output). For each detected peak, the template that has the maximal BOTM output in a small temporal window around the peak is assigned to the spike. The individual processing steps of the method are summarized in Fig. 2. Figure 3 shows a visual comparison of the results of BOTM and other template matching procedures applied to a spike sorting benchmark (Quiroga et al. 2004).

In summary, the method works as follows (Fig. 2): 1. Precomputed matched filters are convolved with the incoming data. 2. Discriminant functions are computed from the matched filter outputs by adding the respective constants. 3. The detection threshold is applied to all discriminant functions. 4. For each threshold crossing, the local maximum of all discriminant functions after the threshold crossing is detected (and before all discriminant functions fall again below the threshold). 5. The temporal position of the peak and the identity of the discriminant function define the identity and time point of the detected spike.

### Resolution of overlapping spikes

If two neurons fire an action potential very close in time, their spike waveforms will overlap in the recording (Fig. 2, right). Overlapping spikes are difficult to detect and classify, since the overlap waveform might be very different from the individual spike waveforms. However, as long as the individual waveforms do not cancel each other in a way that the overlap waveform has virtually zero energy, the individual matched filters do still respond to the overlap. Thus, BOTM will assign the single template with the highest peak to an overlap (which could also be a template not participating in the overlap if the overlap waveform is coincidentally similar to that template) missing at least one of the spikes (Fig. 2, right, ‘BOTM’). To also detect and resolve overlapping spikes one could, in principle, construct all possible overlaps between all available templates with different temporal shifts and add those to the template set. This would, however, dramatically increase the number of templates and introduce two problems: First, with increasing number of templates, each waveform, including noise, can be described by a certain combination of templates. Second, this approach, in a naïve implementation, would be computationally prohibitive. Both problems will be addressed in the following.

For independent spike trains the prior probability to observe an overlap is equal to the product of the individual single-spike prior probabilities (the spike prior). Thus, the more templates are involved in an overlap, the lower is the prior probability for the resulting waveform. Therefore, solutions that feature less templates are naturally favored in our probabilistic framework. With increasing number of templates in an overlap the prior probability to observe such an event decreases, providing a natural cutoff to how many spikes per overlap have to be considered.: At some point the discriminant function for the overlap, *i.e.*,

$$ {d}_{overlap}\left({\xi}_{overlap}\right)={\xi_{overlap}}^T{\boldsymbol{C}}^{-1}{\xi}_{overlap}-\frac{1}{2}{\xi_{overlap}}^T{\boldsymbol{C}}^{-1}{\xi}_{overlap}+ ln\left(p(overlap)\right)<thr $$

will never cross the detection threshold, even for the actual overlap waveform *ξ*_*overlap*_ (see eq. (10) in the appendix). Still, it would be computationally very expensive to compute all convolutions between all (overlap-) templates and the data. However, this is not necessary, since the overlap BOTM discriminant functions (*i.e.,* the BOTM output *d*_*i* + *j*_^*τ*^ for an overlap between template *i* and *j* with temporal shift *τ*) can be directly computed from the individual spike discriminant functions by

$$ {d}_{i+j}^{\tau }(t)={d}_i(t)+{d}_j^{\tau }(t)-{\xi_i}^T{\boldsymbol{C}}^{-1}{\xi}_j^{\tau } $$

where *d*_*j*_^*τ*^(*t*) and *ξ*_*j*_^*τ*^ are the temporally shifted discriminant function and template of neuron *j*, respectively (see Fig. 2, right, ‘Option 1’ and eq. (10) in the appendix for the derivation). Depending on the number of spikes per overlap and their maximal temporal shift considered, this approach can still be computationally expensive.

Therefore, here, we employ a greedy approach. We assume that at least one of the single-spike BOTM outputs will cross the threshold for each overlap and that its peak is giving approximately the correct position of the template in the data. Then, we can subtract the expected influence of this spike from the other discriminant functions, a process which is also referred to as subtractive interference cancellation (SIC, see Fig. 2, right, ‘Option 2’ and eq. (12) in the appendix for the derivation). Fortunately, under the assumption that at least one single-spike discriminant function crosses the threshold for each overlapping spike, we only need to compute the overlap discriminant functions in temporal periods around threshold crossings of single-spike discriminant functions.

### Noise Covariance Matrix

The noise covariance matrix ***C*** plays a crucial role for the Mahalanobis distance, the matched filter and BOTM as well as for some spike sorting procedures (Franke et al. 2010; Marre et al. 2012; Pillow et al. 2013; Pouzat et al. 2002; Shoham et al. 2003). In all cases its inverse is needed. However, the noise covariance matrix can be badly conditioned, *i.e.*, it might have eigenvalues that are close to zero, which makes its inversion unstable. A standard procedure to invert ill conditioned covariance matrices is diagonal loading or shrinkage (Hiemstra 2002; Van Trees 2002): A target covariance matrix ***C***_*T*_, often the identity, is added or merged to the original covariance matrix. We decided to chose the diagonal of ***C***, ***C***_*D*_ = *diag*(***C***) as the target and blended it with the original matrix according to ***C***_*L*_ = *α* ***C*** + (1 − *α*)***C***_*T*_. We noticed that the choice of *α* influenced performance mainly through the temporal length of the resulting filter responses to spikes. With decreasing *α* the filters become more similar to a narrow bandpass filter which could lead to oscillating filter responses. Oscillations in the filter outputs were not a problem for benchmark 3 BOTM + SIC (see below, SIC iteratively subtracts the filter responses to spikes from the filter outputs, therefore removing all oscillations). We did not try to optimize diagonal loading but simply chose *α* = 0.5. For benchmark 3, BOTM + SIC we chose an *α* so that the maximal eigenvalue of ***C***_*L*_ divided by its minimal eigenvalue (*i.e.,* the condition number of ***C***_*L*_) was not larger than 10,000 to ensure that ***C***_*L*_ was invertible.

The noise covariance matrix can be either computed on pieces of noise, that is, periods of data where no spikes were detected (*e.g.*, as in (Marre et al. 2012; Pouzat et al. 2002; Rutishauser et al. 2006)), or on residuals of the spikes after subtracting the templates (*e.g.*, (Pillow et al. 2013; Shoham et al. 2003)). Since the residuals may contain additional variability, *e.g.*, from mis-alignment of the templates (the correct template was subtracted at a wrong position), clustering errors (the wrong template was subtracted), or variability in the neuronal signal (the correct template does not fit perfectly with the occurring waveform), we chose to compute ***C*** on stretches of noise. This has the side-effect that we do not run the risk of overfitting ***C*** to the spike waveforms.

If *T* is the length of the templates (measured in samples) and *N* is the number of recording electrodes, the dimensionality of ***C*** is *T* ⋅ *N* times *T* ⋅ *N*, which can be very large and thus difficult to estimate. However, ***C*** has a special block structure (Pouzat et al. 2002):

$$ \boldsymbol{C}=\left(\begin{array}{ccc}\hfill {C}_{1,1}\hfill & \hfill \cdots \hfill & \hfill {C}_{N,1}\hfill \\ {}\hfill \vdots \hfill & \hfill \ddots \hfill & \hfill \hfill \\ {}\hfill {C}_{1,N}\hfill & \hfill \hfill & \hfill {C}_{N,N}\hfill \end{array}\right) $$

where the blocks *C*_*i*,*k*_ are the covariance matrices between electrode *i* and *k* with *C*_*i*,*i*_ = *C*_*i*,*i*_^*T*^ and *C*_*i*,*k*_ = *C*_*k*,*i*_^*T*^. Each block is a Toeplitz matrix of the cross-correlation function of electrode *i* and *k*. Therefore, we did not estimate ***C*** by directly computing the covariance matrix from *T* ⋅ *N* dimensional snippets of noise, where it would not be guaranteed that (*C*_*i*,*k*_)_*m*,*n*_ = (*C*_*k*,*i*_)_*n*,*m*_ and (*C*_*i*,*k*_)_*m*,*n*_ = (*C*_*i*,*k*_)_*m* + 1,*n* + 1_, but by constructing ***C*** from the respective auto- and cross-correlation functions. This procedure reduces the number of free parameters that need to be estimated to *T* ⋅ *N*, which again reduces the risk of overfitting.

### Evaluation metrics

We evaluated the spike detection and classification performance of the template matching procedures on two different data sets described below. Both were preprocessed in a similar way: The available ground truth information was used to cut true spikes from the recording and to construct the templates. This avoided the problem of aligning the spikes to compute the templates. The template matchers were evaluated for detection and classification performance separately.

For the detection task the template matching output distribution was computed for all spikes as well as for pieces of noise. For each detector the detection error was defined as

$$ \mathrm{Detection}\ \mathrm{Error}=\mathrm{F}\mathrm{P}+\mathrm{F}\mathrm{N} $$

where FP is the number of false positive noise detections and FN is the number of false negative misses. Then, the overlap of the template matching response distributions to noise and spikes was computed and, except for BOTM, the optimal threshold (which minimized the detection error) was estimated numerically. The alternative would have been to compare receiver operator characteristic curves, but BOTM directly provides a threshold. Therefore, we decided to compare its performance to the optimal performance of the other methods.^1^ To detect spikes in the BOTM output a prior probability to observe a spike needs to be chosen. Optimally, this probability is different for each neuron and depends on its firing rate. However, for the benchmarks used in this study, the performance was insensitive to the exact choice of the priors (see Fig. 5). Therefore, instead of choosing a prior for each neuron independently, we assumed them to be equal and set the prior for noise to 0.99 (*i.e.*, we assumed it 100 times more likely to observe noise than a spike).

The sensitivity, *i.e.*, the detection performance at the optimal threshold was defined as

$$ \mathrm{Sensitivity} = 100\frac{\mathrm{TP}}{\mathrm{TP}+\mathrm{F}\mathrm{N}}, $$

which denotes the percentage of correct spike detections (TP) divided by the total number of spikes. Note that this quantity indirectly includes the number of FPs since we minimized FP + FN to find the optimal threshold. In this study we use two different data sets (see below) that differ in the availability of ground truth data: for one data set we know the spike times of all neurons (and, therefore, also the number of neurons), while, in the other data set, spike times are known only for one neuron and it is unknown how many more neurons have been recorded from. For this reason, it is not possible to use the same evaluation metric for both data sets. We, therefore, describe the data-set-dependent evaluation metrics in the following section, together with the applied benchmarks.

- Benchmark 1 (Q): Evaluation on simulated data with full ground truth
- The proposed template matching was evaluated on the publicly available spike sorting benchmark data set described in (Quiroga et al. 2004) which we will refer to as benchmark 1 (Q). This data set was already used by other researchers for spike sorting evaluation (see, *e.g.*, (Bestel et al. 2012; Ghanbari et al. 2009; Herbst et al. 2008)). The data set consists of 4 sub benchmarks labeled “Easy1” to “Diffcult2”. Every sub benchmark consists of 4 different data files (with the exception of “Easy1” which has 8) with decreasing signal-to-noise ratios. All files contain 60s of a simulated single electrode extracellular recording with 24 kHz sampling rate and 3 simulated neurons. Templates and the noise covariance matrix were calculated using the available ground truth information. Short periods of simulated data of length *L* = 61 samples starting 15 samples before the given spike time points were cut and averaged to create the templates. In this benchmark, the ground-truth spike times do not indicate the position of the peak of the spike waveforms in the data but rather their onset. Therefore, we corrected the original spike times by shifting the complete spike train of each neuron by a constant number of samples. This shift yielded a ground truth that reflected the peak positions of the spikes in the data (a feature also a spike sorter without any knowledge of the ground truth could estimate), not their onset. The template matching outputs for the different template matching procedures were computed on the noisy spike waveforms present in the data that were aligned with the corrected ground truth information. Each spike was assigned to the template with the maximal response. Spikes that were correctly assigned were counted as TP while spikes assigned to the wrong template were counted as classification errors CN. The quantity CN relies on knowing the full ground truth, that is, the exact spike times for all neurons. We used the following quantities for the performance comparison:
  $$ \mathrm{Classification}\ \mathrm{Performance}=100\frac{\mathrm{TP}}{\mathrm{TP}+\mathrm{C}\mathrm{N}} $$
- We chose to weight detection and classification equally, combining them in a final performance score of:
  $$ \mathrm{Total}\ \mathrm{Performance}=\frac{\left(\mathrm{Sensitivity}+\mathrm{Classification}\right)}{2}. $$
- Benchmark 2 (H): Evaluation on real recording with partial ground truth
- This data set is the part of the hc-1 data set described in (Henze et al. 2000) and publicly available under http://crcns.org/ and was already used for evaluation of spike sorting algorithms (see, *e.g.*, (Ekanadham et al. 2014; Harris et al. 2000; Schmitzer-Torbert et al. 2005)). The following files were used: d11221.002, d11222.001, d12821.001 and d14521.001. We chose the files depending on the quality of the intracellular recording, *i.e.*, the ones where the intracellular recording showed clearly visible and easily detectable spikes during the whole recording. Each data file consists of several minutes of simultaneous intra- and extracellular recordings in rat hippocampus with a sampling rate of 20 kHz. Ground truth information was available for only one single neuron, extracted from the respective intracellular recording. The extracellular recordings were high pass filtered at 300Hz.
- Spike sorting using mean-shift clustering (Marre et al. 2012) was performed in the space of the first 6 principal components after prewhitening to estimate the templates. The sorting was not optimized manually. The template of the cluster whose spikes best matched the ground truth was used to estimate the performance of the template matching procedures and will be referred to as the “target template”. For all data files the target template was very similar to the template that can be obtained by using only the spikes given by the ground truth, *i.e.*, in all cases spikes from one of the clusters matched the ground truth well enough to get good template estimation.
- For the classification task, the template matching output was computed for all templates and all spikes of the ground truth neuron, as well as for all other spikes detected during the spike sorting. Spikes of the ground truth that were correctly matched to the target template were counted as true positives (TP). Since we do not have the full ground truth for this data set, the quantity CN from the previous benchmark cannot be computed for all neurons. We therefore counted spikes of the target neuron that were falsely assigned to a putative other neuron as CNt. Spikes which were found by the automatic spike sorting procedure which belong to putative other neurons and which were correctly not assigned to the target neuron were counted as true negatives (TN). Spikes not included in the ground truth that were assigned to the target template were counted as false positives (FP). How many of the spikes that actually belong to the target neuron were also classified correctly? This is the classification performance.
  $$ \mathrm{Classification}\ \mathrm{Performance}=100\frac{\mathrm{TP}}{\mathrm{TP}+\mathrm{C}\mathrm{N}\mathrm{t}} $$
- And of all putative spikes detected in the recording that do not belong to the target neuron, how many were correctly classified as noise or spikes from other neurons? This is the specificity (the performance in rejecting spikes that do not belong to the target neuron).
  $$ \mathrm{Specificity}=100\frac{\mathrm{TN}}{\mathrm{FP}+\mathrm{T}\mathrm{N}} $$
- Many different ways of combining the different performance measures into a final score are possible; however, here, we decided to combine them with equal weight into a final score, the total performance.
  $$ \mathrm{Total}\ \mathrm{Performance}=\frac{\left(\mathrm{Sensitivity}+\mathrm{Classification}+\mathrm{Specificity}\right)}{3} $$
- Benchmark 3 (Q): Online template matching of full recording
- To compare the performance of BOTM to the spike sorting performance reported in (Quiroga et al. 2004), we did not use the ground truth to cut perfectly aligned spikes (but the ground truth was used to compute the correct templates). Instead, BOTM was run on the whole data and spikes were detected in the template matching output.
- We used the same data as in benchmark 1 (Q) to evaluate the performance of BOTM as a refinement tool for spike sorting procedures. For this, the performance of BOTM using the correct templates was compared to the performance of a clustering based spike sorting. We chose the spike sorter “wave_clus” described in (Quiroga et al. 2004). “wave_clus” detects spikes using a voltage threshold. The threshold is computed *via* the median absolute deviation (MAD) of the data which is, in the presence of spikes, a more robust way to estimate the standard deviation of the noise. Then, wavelet coefficients are computed for each spike. A subset of the wavelet coefficients is chosen which, putatively, carries most information about the identity of the spikes. Those coefficients are then clustered by using superparamagnetic clustering, a clustering method developed in the context of statistical mechanics and based on simulated interactions between each data point and its K-nearest neighbors.
- For BOTM, the correct templates and the noise covariance matrix were computed from the data using the available ground truth information. To be more specific, ground-truth spike trains were used to cut short pieces out of the data around the true locations of the spikes. These pieces were averaged to calculate the templates. Therefore, the (‘correct’) templates contain a realistic amount of noise due to the averaging process. Pieces of data in which no spikes were present were used to compute the noise auto-covariance function from which the noise covariance matrix was estimated. As a consequence, also the noise covariance matrix was noisy due to the estimation process on a limited amount of noisy data. The optimal filters were computed and convolved with the whole extracellular recordings. The discriminant functions were then thresholded with the optimal threshold (see eq. (9) in the appendix). For each period during which at least one discriminant function was above the threshold, the peak of the maximal discriminant function was detected. The identity of the discriminant function was used as the neurons’ identity and the position of the peak as the time of the spike. This way, detection, alignment and classification were implemented in a single operation. To avoid a second detection of the same peak due to multiple peaks of the filter response, peaks within 8 samples (.33 ms) were compared and only the maximum was kept. Thus, overlapping spikes within this time window could not be resolved. The BOTM + SIC method was evaluated in the same way but, additionally, once a spike was found in the data and assigned to a neuron, the expected filter response to the neuron’s template was subtracted from the discriminant functions (eq. (10) in the appendix) and the detection step was repeated.

## Results

### Performance for individual spikes

**Fig. 6:**
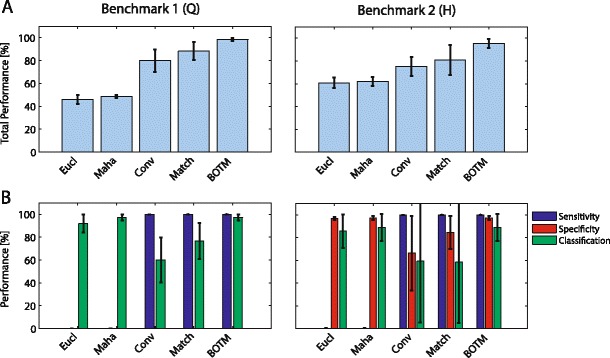
Performance evaluation benchmark 1 (Q) (*left column*) and benchmark 2 (H) (*right column*). *Error bars* indicate standard deviation among different data files of the same benchmark. **a** Total performance of different template matching procedures and **b** Performance for different error categories defined in section “Evaluation metrics”

Figure 6 shows a performance comparison of the presented template matching procedures (Benchmark 1 and 2). While *D*_*i*_^*XC*^(*t*) and *D*_*i*_^*Match*^(*t*) give nearly 100% sensitivity in all cases, sensitivity of *D*_*i*_^*Euc*^(*t*) and *D*_*i*_^*Maha*^(*t*) is close to zero. For these two methods, the respective detector output distributions for spikes and noise are strongly overlapping. Lowering the number of misses (FN) thus strongly increases the number of false positive detections. The optimal threshold is therefore so high, that nearly no spike is detected at all. For classification *D*_*i*_^*Euc*^(*t*) and *D*_*i*_^*Maha*^(*t*) have a consistently high performance while *D*_*i*_^*XC*^(*t*) and *D*_*i*_^*Match*^(*t*) perform poorly. The classification performance of *D*_*i*_^*XC*^(*t*) and *D*_*i*_^*Match*^(*t*) has a high variance since for some files, the optimization procedure to find the optimal threshold was able to set it so high that only spikes of a single neuron were found. For data files in which the target template is indeed the template with the highest amplitude, these procedures have thus near perfect performance. The performance of *D*_*i*_^*XC*^(*t*) and *D*_*i*_^*Match*^(*t*) for data files in which the target template had a small or intermediate amplitude is significantly worse.

The results suggest that subtractive template matching, in the way we used it here, is basically useless to detect spikes. The bad performance can be remedied to a certain degree by limiting the procedure to only templates with large amplitude and by reducing the dimensionality of the templates through either restricting the number of channels, *e.g.*, to one, or shortening the templates. Reducing the dimensionality helps because the main reason for the low sensitivity is the influence of the high dimensional noise on the waveform which is over-proportionally diminished by removing dimensions. However, there is an optimal way to reduce the influence of high dimensional noise and increase the signal-to-noise ratio: the matched filter.

BOTM increases the average performance from below 60% to over 95% with respect to Euclidean distance template matching. If only the classification performance is considered, using Mahalanobis distance or BOTM increased the average performance with respect to Euclidean distance from 91.8% to 97.2% on benchmark 1 and from 91.8% to 93.9% on benchmark 2. We want to point out that these error numbers are not meant to reflect actual template matching performance and likely overestimate performance. In this benchmark only correctly aligned single spikes were used. A situation hardly realistic for real recordings, since noise and the presence of other spikes make a correct alignment difficult. Furthermore, except for BOTM, detection thresholds were set to optimize performance.

The difference between BOTM and *D*_*i*_^*Match*^(*t*) is a template-dependent additive constant, which depends on the energy of the respective template and its prior probability. Adding this constant to the output of the matched filters allows for distinguishing between neurons by using a simple maximum operation (see Fig. 5c). While the decision boundary of a maximum operation on the matched filter output will cut through the clusters (Fig. 5c, dashed line), BOTM moves the coordinate system so that the identity becomes the optimal decision boundary, similar to what LDA would provide. This explains the increased specificity of BOTM as compared to *D*_*i*_^*Match*^(*t*). However, to also provide high specificity, the matched filters need to distinguish signal from noise. Since all templates usually share similar characteristics, such as frequency content, the outputs between the different matched filters are correlated (elongation of clouds in Fig. 5c), which would not be necessarily expected from clusters in the subspace given by pure LDA.

We conclude that while convolution-based template matching provides high sensitivity, distance-based template matching on well aligned spikes provides good classification performance. BOTM combines both advantages in a single operation, thus avoiding the problem of spike alignment. Furthermore, the performance of its analytical threshold is comparable to the optimal threshold of the other methods.

### Effects of errors in the template set on BOTM performance

**Fig. 7:**
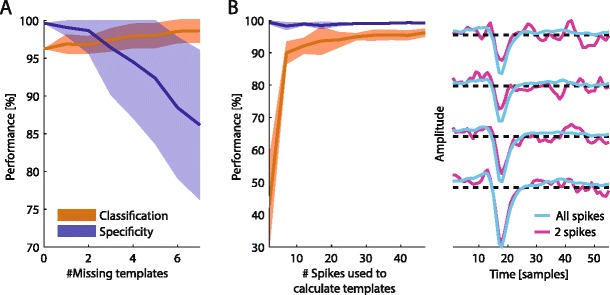
Performance of BOTM with respect to errors in the template set on benchmark 2 (H). **a** The performance of BOTM as a function of the number of templates used for classification. A random subset of templates, excluding the target template was deleted from the set of all templates generated by the initial spike sorting before the BOTM was run. Colored bands indicate one standard deviation. **b** Influence of noise in the templates on BOTM performance. Left panel: For this analysis a random subset of all available spikes for each neuron was used to compute the template. Right panel: A template is shown on all four electrodes, once for using all available spikes (*blue line*) to compute the template and once for using only two spikes (*red line*). For this analysis the same data set as in Fig. 4 was used. Sensitivity is not shown since it was not affected by the errors on the template set

The performance values reported so far have been established under the condition of prior knowledge of the template set. But in how far do errors in the estimation of the templates, *e.g.*, by an initial spike sorting, affect the template matching? A possible error in the creation of template set could be introduced if all spikes of one neuron are missed or falsely classified as noise. In this case, the corresponding template would be missing in the template set. We studied the effect of missing templates on benchmark 2 (H) (the data set also shown in Fig. 4 where 10 templates were found by the initial spike sorting). For the analysis we simply removed a random selection of templates from the template set while always keeping the template assigned to the corresponding target neuron. Figure 7a shows the impact of missing templates on classification performance and specificity. While specificity (*i.e.*, the ability of the template matcher to reject spikes from other neurons) strongly decreases with increasing number of missing templates, classification performance (*i.e.*, the number of spikes of the target neuron actually assigned to the correct unit) actually increases. Sensitivity (not shown in Fig. 7) is not affected by missing templates. The three observations can be easily understood: If a template is missing, the spikes that belong to this template will be either assigned to the closest matching template or will be discarded as noise. If the spikes are assigned to the target template, the specificity will decrease. Since this behavior depends on which template is missing, the variability of the specificity is large (blue band in Fig. 7a). In contrast, spikes of the target neuron which were wrongly assigned using the full template set might be correctly assigned using a restricted template set if the corresponding template is missing. In the extreme, all templates expect the target template are missing and thus spikes can only be assigned to the target template increasing classification performance. The fact that sensitivity (*i.e.*, the ability to differentiate between spikes and noise) is not affected by missing templates is due to the fact that the detection threshold for a given unit does not depend on the templates of the other units.

Another possible problem in the generation of the template set can be noise on the waveforms of the templates. Templates are averages of noisy single spike waveforms, and if not enough single spikes are available to build the template, the resulting waveform may be noisy. We therefore decreased the number of spikes used to compute each template. As Fig. 7b shows, the number of spikes used to compute a template has only a minor effect on classification performance and specificity as long as at least 30 spikes are averaged. Using less than 30 spikes will cause the resulting template to be noisy. The noise on the template will be similar only to the subset of spikes that were used to compute the template. Thus, template may be very well suited to detect exactly those spikes but will be bad in generalizing to other spike waveforms (with different noise) of the same neuron.

Interestingly, sensitivity is not affected by noise on the template. The spike detection threshold depends on the templates energy, the noise covariance matrix and the prior probability to observe a spike. Noise on the template increases the template’s energy while the noise covariance matrix and the spike prior probability remain unchanged. Therefore, the template matching will become more conservative: Noisy templates do not become more sensitive to noise but rather less sensitive to spikes.

### Influence of number of templates on BOTM performance

**Fig. 8:**
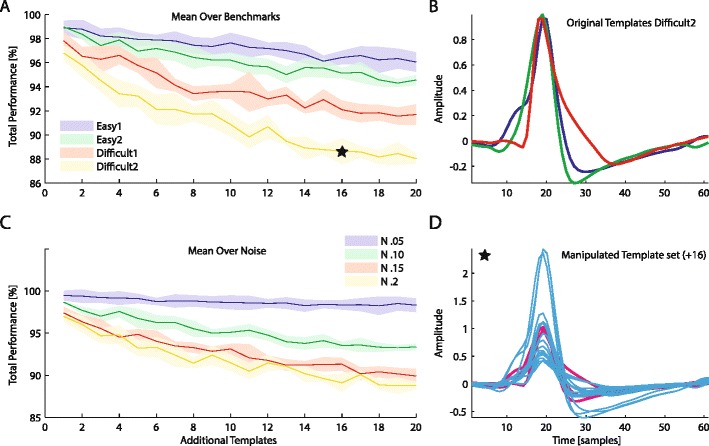
Performance of BOTM with respect to the number of templates on benchmark 1 (**Q**). To the original template set (**b**), a variable number of newly generated templates was added. An example template set with 16 added templates is shown in **d**. New templates are in cyan, the original ones in magenta. New templates were created in a randomized fashion as a linear superposition of existing templates. The added templates were generated in a way to be similar (*e.g.*, with respect to frequency content, peak position *etc.*) but not identical to any of the original templates. **a** Total performance of BOTM for the original 3 templates as a function of the number of added templates, averaged over all noise levels of each data set and obtained with 5 repetitions of template set generation. The star marks the approximate position of the data point given by the template set shown in **d**. **c** Same as **a** but averaged over all benchmarks for each noise level

How sensitive is BOTM to the number of neurons present in the data? Fig. 8 shows the BOTM performance on benchmark 1 (Q) for the original three templates upon adding more templates to the template set. To make the added templates similar to the original three templates in the benchmark, we created them from the original templates: Each new template was either an original template multiplied by a random amplitude *a*_*i*_ or the sum of two such waveforms. This way we created new templates, which were similar to the original 3 templates. However, to avoid that a new template was too smililar to one of the original ones, *a*_*i*_ was chosen from a bimodal probability density function with peaks at 0.5 and 2 respectively:

$$ {a}_i\sim \Big\{\kern1em \begin{array}{c}N\left(2,0.25\right)\kern.2em \mathrm{with}\kern.3em \mathrm{probability}\kern.3em .2\\ {}\kern2em N\left(.5,0.1\right)\kern.2em \mathrm{with}\kern.3em \mathrm{probability}\kern.3em .8\kern1em \end{array} $$

As can be seen from Fig. 8, BOTM performance is negatively affected by adding templates, which reflects the fact that the spike sorting problem becomes more difficult when more templates have to be discriminated. However, the performance does not deteriorate strongly despite the fact, that the templates have similar waveforms. Additionally, the performance saturates for larger number of templates since each additional template is more likely to compete with one of the previously added templates rather than with one of the original three templates.

### Influence of noise covariance matrix

We compared the performance using different noise covariance matrices, namely the original matrix ***C***, only its diagonal and the diagonally loaded matrix. Performance was decreased on average if only the diagonal of ***C*** was used for BOTM, matched filtering and Mahalanobis distance. This is equivalent to assuming the noise to be uncorrelated. Thus, even though the noise in the benchmarks was not necessarily Gaussian, prewhitening did increase classification performance. In all cases *D*_*i*_^*Maha*^(*t*) and BOTM had the highest classification (and rejection) performance while using *D*_*i*_^*Match*^(*t*) and BOTM had the best sensitivity. We did not try to optimize the computation of the noise covariance matrix (*e.g.*, assuming all electrodes to have a similar auto-covariance like done in (Prentice et al. 2011) could be beneficial for multielectrode arrays) and it might be possible to reduce our error rates by a better choice. However, investigating the effect of ***C*** should be preferentially done on real recordings (and potentially for each recording modality individually).

The lower performance of the methods that ignore noise correlations shows that at least for the benchmark data considered here, modeling noise as correlated by taking the covariance structure into account is beneficial for template matching for both detection and classification. However, it should be noted that this is not necessarily true in general, particularly with data sets having strongly non-Gaussian variability due to, *e.g.*, high level multiunit activity, *etc.*

The threshold derived for BOTM is only optimal if the Gaussian noise assumption is valid, if there is no variation of the neurons’ spike shapes, and if there are no changes in its firing rate. To check if these assumptions are violated in a way which would influence the best detection threshold, we also computed the best threshold using the available ground truth in the same way as for the other methods as described in section “Evaluation metrics”. However, we did not find any significant improvement of performance.

We furthermore investigated, how far the assumption of Gaussian noise is fulfilled by the data. Since Benchmark 1 (Q) is simulated data (and thus the noise is artificial), we used the real data from benchmark 2 (H) to estimate in how far pieces of noise follow a Gaussian statistic. Short pieces of 55 samples length were cut out on all 4 channels (thus forming 220 dimensional vectors) in periods of the recordings when the automatic spike detection did not detect any spikes. We then followed the steps of the noise analysis in (Pouzat et al. 2002): First, we estimated the noise covariance matrix. To avoid over fitting we computed the covariance on one portion of the noise pieces and used it to pre-whiten the other portion. Second, we computed the statistics of third moments for random triplets of the 220 dimensions and tested if they were different from 0. We found, in agreement with (Pouzat et al. 2002), that the distribution of energies of the pre-whitened noise pieces follow a *χ* ^2^ -distribution with 219 degrees of freedom, and that the third moment was not significantly different from zero. This indicates that the noise distribution is indeed adequately captured by the noise covariance matrix and the pre-whitened noise pieces follow a multivariate normal distribution.

### Resolution of overlapping spikes and iterative refinement of initial spike sorting

Table 1 shows the performance of BOTM on benchmark 3 (Q). We want to emphasize that the direct comparison of the performance of a blind-clustering-based spike sorting with that of a template-matching procedure that uses the correct templates is difficult. Rather, the results show that using an initial spike sorting first to estimate the templates and then BOTM to (re-)detect and classify spikes could strongly decrease the number of errors, especially for overlapping spikes. The decrease in the error rate is achieved by two mechanisms. First (center-left columns in Table 1), spikes are detected using the optimal filter outputs which solves the detection and avoids the alignment problem. The filter output maximizes the SNR yielding a better sensitivity, and its peak is a good feature to align waveforms. Taking its position and template identity to classify the spikes is equivalent to aligning all waveforms on the respective peaks and performing standard template matching like in benchmarks 1 and 2. Second, BOTM is able to resolve overlapping spikes. According to the criterion in (Quiroga 2004), *i.e.*, a maximal time difference of 64 samples, there are 790 overlapping spikes per data set on average in benchmark 3 (Q). The center-left columns in Table 1 show that most of those overlaps are correctly resolved by BOTM since there are significantly less errors than overlapping spikes. This is due to the fact that, using *α* = 0.5 for diagonal loading, the peaks in the filter outputs are narrower than the original spikes were and, thus, spike waveforms that were overlapping in the original recording might not lead to overlapping peaks in the filter outputs (the effect of *α*, especially on noise robustness, in a similar context is discussed in (Vollgraf et al. 2005)). Those overlaps that were close enough in time to cause simultaneous peaks in the filter outputs could not be resolved by a simple threshold operation on the BOTM filter outputs. Using SIC, however, most spikes were correctly detected and classified (Table 1, center-right columns) significantly reducing the error rate. Therefore, BOTM is a useful method for refining an initial sorting and in the context of online applications where only the beginning of the experiment is sorted offline, *e.g.*, using a clustering procedure. The potential in refining an initial clustering-based spike sorting by BOTM can be seen in the rightmost columns in Table 1. Here, we used the output of the automatic “Wave_clus” sorting (left-most columns) to initialize BOTM. The resulting templates include errors introduced by this spike sorting. For example, in the last data file “Difficult 2, 0.20”, “Wave_clus” found only two of the three clusters, thus missing out on one unit. The resulting template matching with only two templates detected most of the spikes of the missing third unit and assigned them mainly to one of the other units. Overall, BOTM was able to reduce the error rates on all data files and led to an increase in the over-all performance from 83.9% to 97.5%.

**Table 1:** Performance increase by using BOTM after an initial spike sorting on benchmark 3 (Q)

Method:		Wave_clus			Correct templates + BOTM			Correct templates + BOTM + SIC			Wave_clus + BOTM + SIC		
Error type:		Det	Class	Total	Det	Class	Total	Det	Class	Total	Det	Class	Total
Name	Noise												
Easy 1	0.05	921 (73.8)	1 (100.0)	922 (86.9)	76 (97.8)	2 (99.9)	78 (98.9)	13 (99.6)	4 (99.9)	17 (99.8)	11 (99.7)	2 (99.9)	13 (99.8)
	0.10	236 (93.3)	5 (99.9)	241 (96.6)	45 (98.7)	3 (99.9)	48 (99.3)	5 (99.9)	2 (99.9)	7 (99.9)	4 (99.9)	2 (99.9)	6 (99.9)
	0.15	374 (89.2)	5 (99.9)	379 (94.5)	59 (98.3)	5 (99.9)	64 (99.1)	5 (99.9)	0 (100.0)	5 (99.9)	8 (99.8)	0 (100.0)	8 (99.9)
	0.20	999 (71.2)	12 (99.7)	1011 (85.4)	61 (98.2)	9 (99.7)	70 (99.0)	9 (99.7)	3 (99.9)	12 (99.8)	9 (99.7)	3 (99.9)	12 (99.8)
Easy 2	0.05	174 (94.9)	3 (99.9)	177 (97.4)	51 (98.5)	19 (99.4)	70 (99.0)	2 (99.9)	1 (100.0)	3 (100.0)	2 (99.9)	2 (99.9)	4 (99.9)
	0.10	193 (94.5)	10 (99.7)	203 (97.1)	39 (98.9)	21 (99.4)	60 (99.1)	7 (99.8)	1 (100.0)	8 (99.9)	6 (99.8)	2 (99.9)	8 (99.9)
	0.15	184 (94.6)	45 (98.7)	229 (96.6)	43 (98.7)	36 (98.9)	79 (98.8)	4 (99.9)	2 (99.9)	6 (99.9)	4 (99.9)	6 (99.8)	10 (99.9)
	0.20	637 (81.9)	306 (91.3)	943 (86.6)	85 (97.6)	93 (97.4)	178 (97.5)	6 (99.8)	2 (99.9)	8 (99.9)	6 (99.8)	7 (99.8)	13 (99.8)
Difficult 1	0.05	274 (91.9)	0 (100.0)	274 (96.0)	37 (98.9)	17 (99.5)	54 (99.2)	3 (99.9)	17 (99.5)	20 (99.7)	2 (99.9)	18 (99.5)	20 (99.7)
	0.10	201 (94.2)	41 (98.8)	242 (96.5)	39 (98.9)	50 (98.5)	89 (98.7)	18 (99.5)	11 (99.7)	29 (99.6)	18 (99.5)	12 (99.7)	30 (99.6)
	0.15	217 (93.8)	81 (97.7)	298 (95.7)	46 (98.7)	132 (96.2)	178 (97.4)	11 (99.7)	17 (99.5)	28 (99.6)	9 (99.7)	17 (99.5)	26 (99.6)
	0.20	405 (88.1)	651 (80.9)	1056 (84.5)	45 (98.7)	278 (91.9)	323 (95.3)	19 (99.4)	10 (99.7)	29 (99.6)	20 (99.4)	10 (99.7)	30 (99.6)
Difficult 2	0.05	183 (94.6)	1 (100.0)	184 (97.3)	45 (98.7)	34 (99.0)	79 (98.8)	9 (99.7)	8 (99.8)	17 (99.7)	8 (99.8)	9 (99.7)	17 (99.7)
	0.10	157 (95.5)	8 (99.8)	165 (97.6)	33 (99.0)	38 (98.9)	71 (99.0)	4 (99.9)	7 (99.8)	11 (99.8)	5 (99.9)	9 (99.7)	14 (99.8)
	0.15	193 (94.4)	443 (87.1)	636 (90.8)	51 (98.5)	153 (95.6)	204 (97.0)	5 (99.9)	8 (99.8)	13 (99.8)	8 (99.8)	9 (99.7)	17 (99.8)
	0.20	492 (85.9)	1462 (58.1)	1954 (72.0)	104 (97.0)	386 (88.9)	490 (93.0)	7 (99.8)	14 (99.6)	21 (99.7)	36 (99.0)	1124 (67.8)	1160 (83.4)
Total		5840 (89.4)	3074 (94.4)	8914 (83.9)	859 (98.4)	1276 (97.7)	2135 (96.1)	127 (99.8)	107 (99.8)	234 (99.6)	156 (99.7)	1232 (97.8)	1388 (97.5)

Numbers indicate absolute numbers of respective errors, numbers in brackets indicate performance as defined for benchmark 1 (Q) in percent, rounded to one decimal digit. Spike sorting errors for “Wave_clus” as reported in reported in (Quiroga et al. 2004) (leftmost columns), the proposed methods BOTM (center-left columns), BOTM with subtractive overlap resolution (SIC) (center-right columns, and BOTM + SIC on using the result of “Wave_clus” as an initialization (rightmost columns), are given. “Det” are detection errors, “Class” classification errors and “Total” their sum. Values in column “Noise” indicate the standard deviation of the noise relative to the peak of the templates. For “Wave_clus” the errors were estimated independently for detection and classification. Classification errors were computed using all spikes in the data. Thus, spikes that were not detected were still used for the classification task. For BOTM the spike detection and classification were done in the same step without using the ground truth to align the spikes. Spikes that were not detected were also not classified. On the dataset “Difficult 2, 0.20” “Wave_clus“found only 2 of the three units. This is reflected in higher error rates for “Wave_clus + BOTM + SIC” since one template was missing for the template matching

## Discussion

We analyzed the performance of different template matching procedures. We showed that distance based template matching is not suitable to detect spikes while the performance of convolution-based template matching for classifying spikes may be low depending on the templates in the data. For the case of colored Gaussian noise we use a Bayesian approach to derive an optimal template matching. The proposed BOTM algorithm we show to outperform the other methods on a number of benchmark data sets. The probabilistic framework provides a robust way to resolve overlapping spikes, even in the presence of a relatively large number of templates (see Fig. 4 where 10 templates are present). Since the BOTM procedure is fast and computationally simple it is also suitable for hardware implementation and potential real-time applications. BOTM can in principle be applied to all detection and classification problems which include linear separation of multiple transient signals. Furthermore, there is no need to align the spike waveforms before classification, a step that is usually error-prone.

The probabilistic approach and the solution derived here is related to earlier work (Pillow et al. 2013) but exhibits some important differences. Our approach shows the connection between LDA and matched filtering, and can be implemented by linear filters. This can be an important advantage for hardware implementations and closed-loop experiments, since our method is online-capable. This allows for applying adaptive template matching strategies, where the templates and filters are gradually adapted over time depending on the previously found spikes. We did not attempt using an adaptive strategy in this study (see discussion below). The method presented in (Pillow et al. 2013) solves a global optimization problem, while we solve the overlap problem locally in time. Our approach could entail a slight increase in sorting errors in those cases where future spike classifications are important to make the correct decision (resolving an overlapping spike from the future backwards rather than from the past onwards), but it offers the advantage of having an online algorithm and faster classification. Furthermore, the locality reduces the dimensionality of the noise covariance matrix in the range of the template length, not the length of the recordings, which makes it feasible for us to estimate the noise covariance matrix on short periods of noise. The weights of the pros and cons of the methods largely depend, however, on the experimental context.

### Non-stationary templates

Spike waveforms from neurons are known to vary on two different time scales: In the range of milliseconds (Fee et al. 1996), depending on the time between to spikes of the same neuron, and on a larger time scale owing to a movement of the neurons with respect to the electrodes (Franke et al. 2010). Both sources of variability, but prevailingly the movement-induced variability, can be addressed by using adaptive filters. The benchmarks used in this study are, due to a lack of strong waveform variability, however, not well suited to test an adaptive approach, so that further work will be necessary to investigate the potential of adaptive strategies.

### Noise covariance matrix and gaussian noise assumption

BOTM assumes noise to be multivariate colored Gaussian. This was found to be a good description of real noise (Pouzat et al. 2002), but, other studies claim that the distributions of spike waveforms are better explained by t-distributions (Shoham et al. 2003). Neither of the data sets used in this study were constructed to follow this assumption. In fact, the noise in the benchmark data set from (Quiroga et al. 2004) was created by copying many templates with small amplitudes into the data, but it should be also noted that this dataset did not include multiunit activity, which is one of the main factors introducing deviations from Gaussian distributions. The data set from (Henze et al. 2000) are real recordings where noise is likely to contain small amplitude spikes from neurons that are further away from the electrodes but our analysis showed that the Gaussian assumption might be well justified. For both data sets using the colored Gaussian noise assumption significantly increased spike detection and classification performance. This is consistent with the observation that although in many classification problems the assumption of normality and of a common covariance matrix among clusters is often violated, linear classifiers assuming colored Gaussian noise still perform surprisingly well (Duda et al. 2001; Li et al. 2006).

Should the main variability of clusters be caused by the neurons, *e.g.,* while bursting, and not by noise, the decision boundaries derived *via* the noise covariance matrix (and not the cluster covariance matrix) might be suboptimal. This could be remedied by using multiple templates per neuron, an approach especially promising for bursting neurons which can produce several distinct, sometimes even non-overlapping clusters.

A question that remains open is in how far the noise statistics (and thus the noise covariance matrix) are stable during an experiment and how well they can be estimated, *e.g.*, for artifacts and ripples (Fig. 1). This will depend strongly on the recording conditions and might vary from setup to setup. However, similar problems were already faced in radar applications (Melvin 2004) and it might be beneficial to determine if those solutions are applicable also to the analysis of extracellular recordings.

### Resolution of overlapping spikes

Several approaches were recently developed to resolve overlapping spikes (Atiya 1992; Ekanadham et al. 2014; Franke et al. 2010; Lewicki 1994; Marre et al. 2012; Pillow et al. 2013; Prentice et al. 2011; Segev et al. 2004; Vargas-Irwin and Donoghue 2007; Wang et al. 2006; Zhang et al. 2004). Most of them are based either on a greedy iterative subtraction scheme to remove spikes and detect overlaps (*e.g.*, (Marre et al. 2012; Segev et al. 2004)) or on searching the best fit in the space of all possible overlaps (*e.g.*, (Pillow et al. 2013; Prentice et al. 2011)). Ekanadham and co-authors (2014) suggest to resolve overlapping spikes while performing clustering. The method we propose here is similar to several of the abovementioned approaches: the brute-force (Option 1 in Fig. 2) and the iterative subtraction scheme (Option 2, SIC). In contrast to the other methods, however, BOTM performs the overlap resolution using the filter outputs of the matched filters instead of the original recorded data. This increases the discriminability of spike waveforms from different neurons and reduces the influence of noise. Although our method is computationally less expensive it still yields comparable results to (Ekanadham et al. 2014) on Benchmark 3.

## Appendix

Here we give the derivation of the BOTM procedure. The derivation shows that applying linear discriminant analysis (LDA) to each sample of the data is equivalent to computing the matched filter output for each template and adding a template-dependent constant. First, we review the matched filter (Choi 2010; Kay 1998; Van Trees 2002). Then, we introduce LDA (Fisher 1936) from the perspective of a classification problem. We continue by recognizing that if the vectors, classified by LDA, are thought to be short pieces of data beginning at each possible temporal position, the linear discriminant functions for each class (*i.e.*, the BOTM filter outputs for each template) can elegantly be computed from the matched filter outputs. From the probabilistic generative model underlying LDA we can also derive the theoretically optimal threshold for spike detection in the BOTM outputs. Finally, we note that to detect and classify an overlapping spike, the discriminant function for the overlap can be computed from the individual discriminant functions by summation with a temporal shift. This offers two possibilities to resolve overlapping spikes, namely the computation of all discriminant functions for overlapping spikes with different shifts or a greedy subtractive procedure.

## Matched filtering

If the waveform *ξ* of a transient signal embedded in a noisy recording is known, the FIR filter which maximizes the filter response $y(t)={\displaystyle \sum_i{f}_i*}x(t)$ to *ξ* while minimizing the response to noise is called the matched filter. The response of the filter to the template is given by *y* = *f* ^*T*^ *ξ* while its expected response to Gaussian noise *η* ∼ *N*(0, *C*) is given by *y*(*t*) = *E*[*f* ^*T*^ *η*]. Thus, the signal-to-noise ratio can be defined as

$$ SNR=\frac{{\left|{f}^T\xi \right|}^2}{E\left[{\left|{f}^T\eta \right|}^2\right]} $$

The filter that maximizes the SNR with is then given by (for a derivation see, *e.g.*, (Van Trees 2002) and in the context of spike sorting (Franke 2011))

$$ f=\frac{C^{-1}\xi }{\beta } $$

where *β* is a normalization constant.

## Linear discriminant analysis

Consider a multi-class classification problem and a new unlabeled data point *x*. To which class should we assign *x*? If we want to minimize the classification error, we will assign *x* to the class with maximal conditional probability *p*(*i*|*x*), *i.e.*, assign *x* to class *i*_*opt*_ if

1 $$ {i}_{opt}=\begin{array}{c}\hfill \arg\;\max\;p\left(i\left|x\right.\right)\hfill \\ {}\hfill i\hfill \end{array} $$

Using Bayes rule one obtains:

2 $$ {i}_{opt}=\begin{array}{c}\hfill \arg\;\max \hfill \\ {}\hfill i\hfill \end{array}\frac{p\left(x\left|i\right.\right)p(i)}{{\displaystyle {\sum}_kp\left(x\left|k\right.\right)p(k)}} $$

where *p*(*x*|*i*) is the data likelihood given class *i*. In the context of the classification problem, we are not interested in the exact probabilities but only in the relations between them. We are searching for a discriminant function $\widehat{d}$ that will give us the same classification function as (eq. 1)

3 $$ {\widehat{d}}_i(x)>{\widehat{d}}_j(x)\iff p\left(i\left|x\right.\right)>p\left(j\left|x\right.\right) $$

but omits all unnecessary computations; *e.g.,* the denominator in (2) appears on both sides of the inequality and can thus be ignored. We obtain

4 $$ {\widehat{d}}_i(x)=p\left(x\left|i\right.\right)p(i) $$

If *p*(*x*|*i*) is a multivariate normal distribution $p\left(x\Big|i\right)=\frac{1}{{\left(2\pi \right)}^{\frac{k}{2}}{\left|{\boldsymbol{C}}_i\right|}^{\frac{1}{2}}}{e}^{-\frac{1}{2}{\left(x-{\xi}_i\right)}^T{{\boldsymbol{C}}_i}^{-1}\left(x-{\xi}_i\right)}$ with mean *ξ*_*i*_ and covariance matrix *C*_*i*_, we obtain

5 $$ {\widehat{d}}_i(x)=\frac{1}{{\left|{\boldsymbol{C}}_i\right|}^{\frac{1}{2}}}{e}^{-\frac{1}{2}{\left(x-{\xi}_i\right)}^T{{\boldsymbol{C}}_i}^{-1}\left(x-{\xi}_i\right)}p(i) $$

where *k* is the dimension of the data *x* and the term ${\left(2\pi \right)}^{\frac{k}{2}}$ has been dropped because it is the same for all classes. Note that in the general case each class can have its own covariance matrix *C*_*i*_.

Taking the logarithm we formally obtain, the discriminant function *d*

$$ {d}_i(x)= \ln {\widehat{d}}_i(x)=-\frac{1}{2} \ln \left(\left|{\boldsymbol{C}}_i\right|\right)-\frac{1}{2}{\left(x-{\xi}_i\right)}^T{{\boldsymbol{C}}_i}^{-1}\left(x-{\xi}_i\right)+ ln\left(p(i)\right) $$

which is called Fisher’s quadratic discriminant function. If we can constrain all classes to share the same covariance matrix *C* = *C*_*i*_, ∀ *i* (homoscedasticity) then the term $-\frac{1}{2} \ln \left(\left|{C}_i\right|\right)$ is again shared by all discriminant functions and can be dropped. Expanding the squared form then yields

6 $$ {d}_i(x)={x}^T{\boldsymbol{C}}^{-1}{\xi}_i-\frac{1}{2}{\xi_i}^T{\boldsymbol{C}}^{-1}{\xi}_i+ ln\left(p(i)\right) $$

which is the function used in linear discriminant analysis (LDA) (Fisher 1936). The result is similar to the M-ary detection case in (Choi 2010), chapter 2.5.

In the context of spike sorting the classes represent different neurons and all vectors are assumed to be perfectly aligned spike waveforms. Then the assumption that all clusters have the same covariance matrix is partly justified: noise in the spike waveforms can be modeled as being independent of the identity of the spike.

## Derivation of Bayes optimal template matching

We now combine the LDA results with the matched filtering procedure. According to the model assumptions, spike templates are centers of multivariate normal distributions with a shared (noise) covariance matrix. If we now interpret each temporal position *t* in a continuous recording *X*(*t*) as a data point and thus as a potential spike, we need to compute the discriminant function of the full continuous recording to all templates.

Linear discriminant functions *d* can be calculated by a filtering the continuous recording with the matched filter *f*_*i*_ = *C* ^− 1^ *ξ*_*i*_ (of eq. (6))

7 $$ {d}_i(t)=X{(t)}^T{f}_i-\frac{1}{2}{\xi}_{{}_i}^T{C}^{-1}{\xi}_i+ ln\left(p(i)\right) $$

where *X*(*t*) is a vector of concatenated multi-electrode data starting at time *t*. The term ${c}_i=-\frac{1}{2}{\xi}_i^T{C}^{-1}{\xi}_i ln\left(p(i)\right)$ does not depend on *X*(*t*), hence

$$ {d}_i(t)=X{(t)}^T{f}_i+{c}_i $$

The output of the matched filters (apart from an additive constant) can thus directly be interpreted as the Fisher discriminant function.

## Discriminant function for noise

Obviously, not every sample of the data corresponds to a spike. The problem to decide whether a given sample is noise instead of a spike can be solved by introducing a “template” (and thus a discriminant function) for noise. For zero mean noise we introduce the null vector 0 as the noise template n and obtain the corresponding discriminant function

8 $$ {d}_n(t)\Big\rangle X{(t)}^T{C}^{-1}0-\frac{1}{2}0{C}^{-1}0+ ln\left(p(n)\right) $$

where *p*(*n*) is the prior probability for a given sample of data to be noise (potentially including spikes from neurons for which no template is given, *e.g.*, multi-unit activity). With $p(n)=1-{\displaystyle \sum_ip(i)}$, we obtain

9 $$ {d}_n(t)\Big\rangle thr={d}_n= \ln \left(1-{\displaystyle \sum_ip(i)}\right) $$

Equation (9) provides a natural threshold for spike detection which will usually be very close to 0.

## Discriminant function for overlapping spikes

The discriminant function for an overlap between spikes from neuron *i* and *j* with time shift *τ* can be defined analogously by introducing a new template *d*_*i* + *j*_^*τ*^(*t*) = *ξ*_*i*_ + *ξ*_*j*_^*τ*^ for the overlap.

*ξ*_*j*_^*τ*^ denotes the template of neuron *j* shifted by time *τ*. We obtain

10 $$ {d}_{i+j}^{\tau }(t)=X{(t)}^T{C}^{-1}\left({\xi}_i+{\xi}_j^{\tau}\right)-\frac{1}{2}{\left({\xi}_i+{\xi}_j^{\tau}\right)}^T{C}^{-1}\left({\xi}_i+{\xi}_j^{\tau}\right)+ ln\left(p(i)p(j)\right) $$

Equation (10) can be rearranged to

11 $$ {d}_{i+j}^{\tau }(t)={d}_i(t)+{d}_j^{\tau }(t)-{\xi_i}^T{\boldsymbol{C}}^{-1}{\xi}_j^{\tau } $$

where *d*_*j*_^*τ*^ is a time shifted version of *d*_*j*_. Equation (11) allows computing the overlap discriminant function from the individual spike discriminant functions without actually constructing the overlap template or computing its filter. This method can be extended to overlaps with more than 2 participating spikes.

## Subtractive interference cancellation

Since the computation of the overlap discriminant functions can still be very expensive, we can save computation time with a greedy subtraction procedure: Once a single spike is detected in the single spike discriminant functions, we can use eq. (11) to update the other single spike discriminant functions to reflect the presence of the already found spike. This is done by subtracting the expected filter response of each filter to the detected spike from the respective discriminant functions, an approach also referred to as subtractive interference cancellation (SIC) (Moshavi 1996). For this subtraction the precise position of the waveform in the data is needed, otherwise, a shifted version might be subtracted, causing a residual error. The original sampling rate of the data might not be fine enough; therefore, it can be advantageous to upsample the data before subtraction.

To update the discriminant function for template *i* if a spike of template *j* was found at time *t*_0_ we set

12 $$ {d}_i\left({t}_0-\tau \right)\leftarrow {d}_i\left({t}_0-\tau \right)-{\xi}_i^T{C}^{-1}{\xi}_i^T+ ln\left(p(j)\right) $$

which follows directly from eq.(11). It changes the value of *d*_*i*_(*t*) to the correct value of *d*_*i* + *j*_^*τ*^(*t*) after subtraction of *ξ*_*j*_ from the data at time *t*_0_. This is the update used in this study, but a possible alternative heuristic would be

13 $$ {d}_i\left({t}_0-\tau \right)={d}_i\left({t}_0-\tau \right)-{\xi_i}^T{\boldsymbol{C}}^{-1}{\xi}_j^{\tau } $$

by ignoring the prior probability *p*(*j*) for spike *j*. This approach effectively removes the influence of spike *j* and repeats the spike detection process without taking into account that a spike was already found, which is identical to subtracting the template from the raw data and re-computing the complete template matching.

## Footnotes

1 Note that this overestimates the performance of the other methods, since in a real situation their optimal threshold is not known.
